# Hand reanimation: functional free gracilis transfer or transfer of the distal tendon of the biceps to the flexor digitorum profundus and flexor pollicis longus as surgical options

**DOI:** 10.31744/einstein_journal/2024AO0719

**Published:** 2024-10-21

**Authors:** Raquel Bernardelli Iamaguchi, Maria Virginia Arranz, Rames Mattar

**Affiliations:** 1 Universidade de São Paulo Institute of Orthopedics and Traumathology Hand Surgery and Reconstructive Microsurgery Group São Paulo SP Brazil Hand Surgery and Reconstructive Microsurgery Group, Institute of Orthopedics and Traumathology, Universidade de São Paulo, São Paulo, SP, Brazil.; 2 Hospital Israelita Albert Einstein São Paulo SP Brazil Hospital Israelita Albert Einstein, São Paulo, SP, Brazil.

**Keywords:** Tendons, Tendon transfer, Upper extremity, Paralysis, Recovery of function, Muscles, Treatment outcome, Hand

## Abstract

**Objective:**

Hand reanimation for finger flexion in patients with total paralysis remains a reconstructive challenge, especially when tendon transfers or neurological reconstruction options are no longer viable. This study aimed to describe a series of patients without hand function by evaluating two hand reanimation techniques.

**Methods:**

This observational retrospective study used a case series of hand reanimation. Two techniques were performed-functional free gracilis muscle transfer with microsurgical reconstruction, and transfer of the recovered biceps to the flexor digitorum profundus and flexor pollicis longus with tendon graft augmentation. The two groups, each undergoing one of the techniques, were evaluated for the final functional results using the British Medical Research Council (BMRC) grading system.

**Results:**

Six consecutive patients with total hand paralysis were included, with a mean final follow-up of 7.5 years. After intervention, two patients, one from each technique group, achieved a BMRC grade 2. In the group where tendon transfer of the biceps to the finger flexors was performed, two patients achieved a BMRC grade 3. Additionally, two patients who underwent functional free muscle transfer were achieved a BMRC grade 4.

**Conclusion:**

The transfer of biceps to the finger flexors using tendon grafts, which involves fewer technical difficulties and reduced demands from the surgical team compared to functional free muscle transfer, is a viable alternative for treating patients requiring hand reanimation. However, functional free muscle transfer is recommended as the first option when technically feasible and adequate donor nerves are available, due to its potential for achieving greater final muscular strength in the finger flexors. Reconstructive microsurgeons can use both techniques as viable surgical options for hand reanimation.

## In Brief

Iamaguchi et al. demonstrated that the transfer of biceps to the finger flexors using a tendon graft is a viable alternative for the treatment of patients requiring hand reanimation, offering fewer technical difficulties and reduced surgical team demands compared to functional free muscle transfer. However, functional free muscle flaps may achieve greater final muscular strength in the finger flexors.

## Highlights

- Hand reanimation for finger flexion during total paralysis remains a reconstructive challenge.
- Few surgical options are available for the late presentation of total paralysis of the hand and wrist.
- Functional freemuscle flap or transfer of the recovered biceps to the long flexors of the digits and flexor pollicis longus may be the last resort.
- Each case should be evaluated individually to select the best surgical option.

## Graphical abstract

**Figure:**
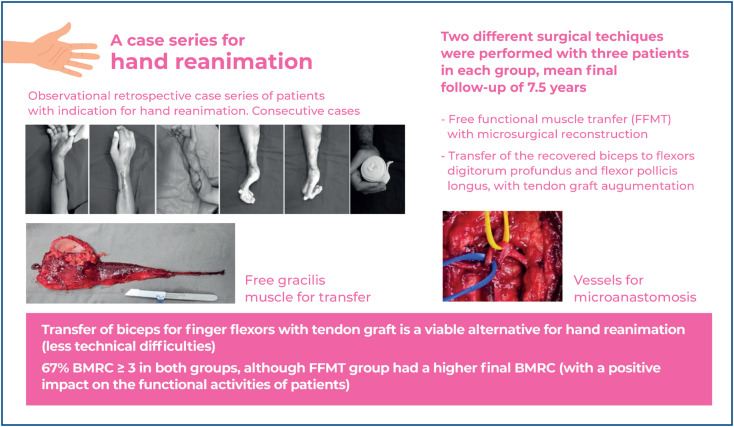

## INTRODUCTION

Hand reanimation for finger and thumb flexion in cases of total paralysis remains a challenge in reconstructive surgery, and the available options for hand reanimation include nerve grafts, distal neurotization for grip function, tendon transfers, functional free muscle transfer, amputation, and prostheses.^(1,2)^ Unfortunately, in many cases of upper limb paralysis, hospital referral for definitive treatment is often postponed. In instances of late presentation of upper limb paralysis, when neurological reconstruction is no longer possible and forearm transfers are not feasible, the functional free muscle flap (FFMF) and transfer of the recovered biceps to the long flexors of the digits and the flexor pollicis longus may be the last option. However, consistent results are not found in the literature.

## OBJECTIVE

To describe a case series of consecutive patients with no hand function due to late presentation of upper limb paralysis or severe avulsion injuries with upper limb amputation.

## METHODS

This was an observational, retrospective study of a case series of patients with indications for hand reanimation. Inclusion criteria were as follows: British Medical Research Council grading system (BMRC) grade 0 resulting from physical examination of the fingers, wrists, and thumb flexion, or no hand function of prehension or pinching. The selected patients were all consecutive patients that could not be treated using tendon transfers or neurological reconstruction because they were not clinically feasible.

Two procedures, functional free gracilis transfer and transfer of the recovered biceps to the long flexors of the digits and flexor pollicis longus, were performed following the standard technique. All surgeries were performed by the first author, who has >15 years of reconstructive microsurgery experience, at the Public Hospital of the *Universidade de São Paulo*, Brazil. The criteria for choosing each technique were as follows: cause of finger and wrist neurological paralysis, elbow flexion strength, and availability of a suitable donor nerve for neurotization in free muscle transfer. All patients signed a consent form prior to surgery, case description, and photographs.

The patients were divided into two groups based on the hand reanimation technique used (functional free gracilis transfer or transfer of the recovered biceps to the long flexors of the digits and flexor pollicis longus), and the functional outcomes were assessed. The functional outcomes of the two groups were evaluated based on the flexion strength of the digits, which was assessed according to the modified BMRC Scale. Results were classified as good if the BMRC grade was ≥3 and bad if the grade was <3.

This study was approved by the local and national research ethics committee of *Hospital das Clínicas, Faculdade de Medicina, Universidade de São Paulo*, under CAAE: 29539719.8.0000.0068; #4.738.060.

## RESULTS

**Figure 1:**
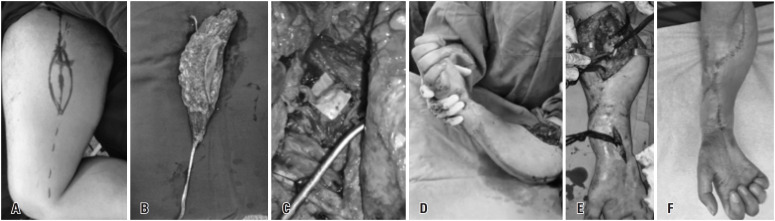
Functioning free gracilis transfer with flap dissection (A and B); neurotization and microanastomoses of finger flexors (C); and non-microsurgical transfer of the biceps distal tendon to the long flexors of the digits and flexor pollicis longus with skin incisions (D and E) and postoperative finger flexion tension (F)

In this study, two groups, each comprising three consecutive patients who underwent different hand reanimation techniques, were included. All surgeries were performed by the first author. The mean follow-up was 7.5 years (Figure 1). Preoperatively, all the patients had a BMRC grade of 0 for finger and wrist flexion. All patients were followed-up at the outpatient clinic and regular occupational therapy sessions to improve functional recovery until maximum finger and thumb flexion was achieved. No patients were excluded from the case series. Donor site morbidity was not reported in patients who underwent functional free muscle transfer (FFMT), with normal ambulation and no reports of pain. Among the patients who underwent biceps transfer, none presented with loss of elbow flexion strength due to the preservation of lacertus fibrosus.

Regarding the functional outcomes related to muscular strength, two patients who achieved a final BMRC of 4 returned to their previous activities; both patients underwent FFMT for finger flexion. Two patients who achieved BMRC grades 3 and 2, respectively, retired from work, including a housewife who initially experienced an industry-related forearm amputation due to a work-related accident. All retired patients reported some form of informal freelance work.

**Figure 2:**
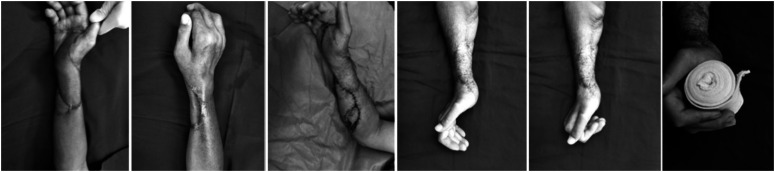
Case 1; The patient underwent superficial circumflex Iliac artery perforator flap; preoperative functional free gracilis and postoperative final finger flexion

Case 1: A 41-year-old male patient with an electric high-voltage shock injury that developed massive soft tissue loss and scarring with wrist contracture. Physical examination initially showed no active finger or wrist flexion. One year post-injury, the patient underwent a Superficial Circumflex Iliac Artery Perforator (SCIP) flap to correct wrist contracture and flexor tenolysis. After correction, the patient demonstrated elbow flexion with BMRC grades 5 and 0 for finger and wrist flexion, respectively, and BMRC grade 2 for finger and wrist extension. As the wrist and finger extensors were not strong enough for transfer, the patient underwent functional free gracilis transfer to the finger flexors, with neurotization of the FFMT using the motor branch of the pronator teres and proximal attachment to the medial epicondyle. At the final follow-up (8 years), the BMRC grade was 4 (Figure 2).

Case 2: A 38-year-old male patient with traumatic total brachial plexus injury who underwent a functional free gracilis flap to restore elbow flexion, achieving excellent results: BMRC grade 4 for elbow flexion and wrist extension to the neutral position, and BMRC grades 3 and 0 for finger and wrist flexion and extension, respectively. Two years after the first surgery, the patient underwent a second-stage functional free gracilis transfer to the finger flexors, with the proximal gracilis attachment at the third and fourth ipsilateral ribs and neurotization of the intercostal nerves at the same level. At the final follow-up (9 years), the BMRC was grade 2 for finger flexion.

**Figure 3:**
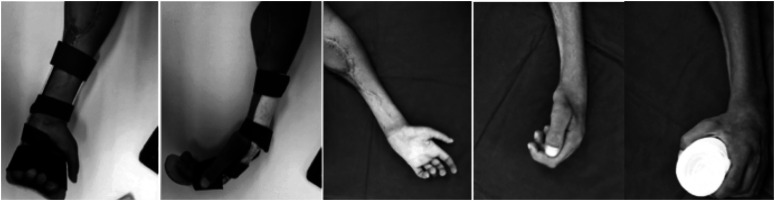
Case 3; postoperative orthoses and final finger flexion results

Case 3: A 17-year-old male patient with a total brachial plexus injury who underwent nerve grafting to the upper and middle trunk. Physical examination revealed BMRC grade 4 for elbow flexion and BMRC grade 0 for finger and wrist flexion and extension. In 2016, a functional free gracilis for finger flexors was performed. At the final follow-up (10 years), the BMRC was grade 4 for finger flexion (Figure 3).

**Figure 4:**
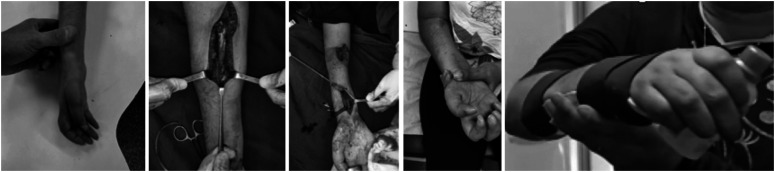
Case 4; The patient exhibited preoperative finger flexion of BMRC grade 3. Intraoperative technique of the transfer of biceps for finger flexion, and final result of finger flexion

Case 4: A 32-year-old female patient with a total brachial plexus injury caused by a motorcycle accident presented with spontaneous recovery of the biceps brachii, but without recovery of the lower trunk. Physical examination showed elbow flexion of BMRC grade 4 and finger and wrist flexion and extension of BMRC grade 0. The patient underwent trapezius transfer surgery for the external rotation of the shoulder three years after the injury. Additionally, after one year of recovery and occupational therapy for passive motion of fingers and wrist, the patient underwent a transfer of the biceps to the flexors digitorum profundus and flexor pollicis longus with a fascia lata graft. At the final follow-up (6 years), BMRC was grade 3 for finger flexion (Figure 4).

Case 5: A 38-year-old male patient with a total traumatic brachial plexus injury caused by a motorcycle accident underwent brachial plexus exploration and microneurolysis of the upper trunk with no neurotization or grafts at another hospital. Subsequently, four years after the surgery, the patient was referred to our hospital for treatment. During the initial examination, the patient presented with spontaneous recovery of the biceps and underwent shoulder arthrodesis using plates and screws. One year after the consolidation of the shoulder arthrodesis, a transfer of the biceps with a semitendinosus graft was performed for finger and thumb flexion. At the final follow-up (5 years), the BMRC was grade 3 for finger flexion.

Case 6: A 24-year-old female patient presented with a right forearm avulsion due to a work-related accident. Macro-replantation was successfully performed. However, after two weeks, skin necrosis developed at the site of microanastomosis. Consequently, the patient underwent an anterolateral thigh (ALT) flap for coverage following debridement of skin necrosis adjacent to the vascular anastomosis. In the early postoperative period after the ALT flap, the patient experienced complications including replantation thrombosis and hand ischemia, which were successfully treated with Fogarty catheter thrombectomy. Upon physical examination, the patient exhibited elbow flexion of BMRC grade 5 and finger and wrist flexion and extension of BMRC grade 0. One year after the replantation, considering the risk of microsurgery, the patient underwent biceps transfer for hand reanimation using a semitendinosus tendon graft. At the final follow-up (7 years), the BMRC was grade 2 for finger flexion.

The outcomes of both techniques were similar, with good results observed in two cases for each technique. However, the gracilis FFMT group achieved a higher final BMRC Score, which positively impacted the functional activities of patients, especially those with muscle strength of grade 4, compared to two patients in the biceps transfer group who had a grade of 3 for finger flexion. Loss of elbow flexion was not observed in any of the patients who underwent biceps tendon transfer for finger flexion, as the technique preserves the insertion of acertus fibrosus (Table 1).

**Table 1:** Description of cases according to the technique and results

Case	Gender	Age (years)	Surgery	Cause of paralysis	Final BMRC grade
1	Male	41	FFMT	Electric Shock	4
2	Male	38	FFMT	BPI	2
3	Male	17	FFMT	BPI	4
4	Male	32	Biceps transfer	BPI	3
5	Male	38	Biceps transfer	BPI	3
6	Female	24	Biceps transfer	Major amputation	2

FFMT, functional free muscle transfer; BPI, brachial plexus injury.

## DISCUSSION

The reanimation of hand function in a paralyzed hand is one of the most challenging reconstructions in hand surgery, and there is still no definitive protocol for treating these serious injuries. Neurological reconstruction with a nerve graft or nerve transfer should be attempted whenever possible. In cases with late presentation or in the absence of recovery after neurological procedures, few options,^(3)^ including the transfer of the biceps to the finger flexors and FFMF, are available. In our cases, the decision of which technique to perform was individualized based on clinical and anatomical evaluation of the availability of donor nerves for neurotization without the need for a nerve graft for FFMT, the presence of good recipient vessels without scarring in the soft tissue, and the arc of movement of the fingers and wrist. In the absence of adequate microvascular conditions (*i.e.*, the number of microvascular surgeries prior to hand reanimation with a higher risk of unavailable good vessels), the recovered biceps were evaluated for transfer, and muscle strength of BMRC grade 4/5 was considered for muscular transfer with a tendon graft.

In 2009, Oberlin et al. described the transfer of the distal tendon of the biceps to the finger flexors,^(4,5)^ and later, in the same year, the technique was demonstrated by Goubier et al.^(6)^ In our cases, good results were obtained with this technique; obtaining finger flexion for bimanual activities of daily living but not for resistance activities, similar to the literature.^(5)^

The transfer of biceps for hand reanimation can be optimized for stability using wrist arthrodesis. Oberlin et al in 2010^(5)^ performed biceps tendon transfer and wrist arthrodesis simultaneously. However, in our cases, we preferred to perform wrist arthrodesis as a second-stage surgery, especially to achieve stability, after functional recovery of the transferred biceps to the fingers and thumb flexors. In cases of FFMT for hand reanimation, wrist arthrodesis is performed as the second-stage surgery, and therefore, the tenodesis effect of the wrist during finger flexion may help patients perform manual activities.

We would like to highlight two technical considerations regarding the transfer of the distal tendon of the biceps to the finger flexors. First, the choice of the tendon graft is important, with the tensor fascia lata recommended as the tendon of choice.^(5,6)^ However, after harvesting, the tensor fascia lata must be prepared as a folded and/or doubled graft, improper preparation of which can lead to a bulking tendon suture and loss of strength. Therefore, an alternative is harvesting the fascia lata as a thin, long strip, which is comparable to the semitendinosus tendon graft that is of similar length and diameter. However, instead of the tensor fascia lata graft, we preferred the use of the semitendinosus tendon autograft as it is a stronger tendon graft and more suitable in size for the transfer. The second technical issue in biceps transfer is the forearm position in the final suture. It is well established that the flexion force of the fingers is stronger in forearm supination^(7)^ due to transfer of the biceps brachii, which is a main supinator.^(8)^ Therefore, we recommend suturing the distal end of the tendon graft to the flexor digitorus profundus and flexor pollicis longus of the thumb with adequate tension, while maintain the forearm in mid-prone position to optimize the dynamic contraction, which aligns with that reported by Goubier et al. elbow in 90° flexion and fingers flexed with a 4 cm pulp-to-palm distance.^(6)^

In 1970, Tamai et al. described the first experimental free muscle transfer.^(9)^ In 1976, Chen et al. described the transfer of the lateral pectoralis major for finger flexion in ischemic Volkmann's contracture and Zuker et al. published good results with FFMT.^(10,11)^ In 1978, Manktelow et al. described FFMT for finger flexion.^(12)^ Since then, few articles have been published on this topic. In 2009, Terzis et al described 38 cases of functional free gracilis transfer for hand reanimation (finger flexion) in a retrospective study beginning in 1981, with the best results observed in cases of neurotization with the spinal accessory nerve.^(13)^

At our hospital, the preferred muscle for functional free transfer is the gracilis because it is long and thin with adequate muscle strength, has consistent neurovascular anatomy, and has ideal excursion and distal tendon length for tendon suture with minimal donor site morbidity. Additionally, the gracilis muscle is less bulky when contracted for finger flexion when compared to the latissimus dorsi, which is the donor muscle preferred by some authors, a choice justified by the results of greater recovery of muscle strength.^(14)^

Another challenge is the selection of the most suitable donor nerve for gracilis neurotization. One of the best indications for hand reanimation with FFMF is Volkmann contracture, as there may be more options for neurotization^(5,6,8,15,16)^ with intrapleural donor nerves at the forearm level, which are technically easier. However, Volkmann's contracture is rarely seen in our hospital due to early diagnosis and prevention of trauma sequelae. Currently, the most common indications for hand reanimation are complete traumatic brachial plexus palsy, which represents a greater challenge for surgeons because only extraplexual nerves, including the intercostal, phrenic, accessory, and rectus abdominis nerves,^(17-19)^ may be available for neurotization. Furthermore, hand reanimation may be a secondary surgery, meaning that some of these extraplexual donor nerves may not be available after the primary reconstructive surgery. In such cases, the intercostal nerves, which adds technical difficulty and increases the surgical time of FFMF, are the only option. Patients must be aware that the results may be worse with extraplexual donor nerves^(20)^ and that nerve grafts may be necessary.

In cases where we performed functional free gracilis transfer for finger flexion, distal neurotization was possible. Two patients exhibited better results as the free muscle was distally attached to the medial epicondyle.^(21,22)^ The patient of Case 3 had previously undergone free gracilis for elbow flexion using the accessory nerve for neurotization and the intercostal nerve as the donor nerve, which provides worse results than using the accessory nerve.^(11)^ Although the last stage of the double free muscle transfer for complete traumatic brachial plexus palsy described by Doi et al^(23)^ requires recovery of elbow flexion and has variable results described in the literature, it may be the only option that provides good functional results with adequate rehabilitation.^(24)^ In case of late presentation of complete traumatic brachial plexus palsy, the authors of a systematic review recommended nerve sparing to an FFMF for better final functional results when compared with nerve reconstruction.^(25)^

Functional free muscle transfer is a highly complex surgery with various technical possibilities that need to be chosen on a case-by-case basis, leading to variable results for hand reanimation. In our study, FFMF achieved a higher final BMRC grade of 4 in two patients, while the transfer of biceps to the finger flexors had the best result, with two patients achieving a BMRC grade 3. Therefore, when a patient is motivated to undergo microsurgery, the clinical conditions are adequate, and good recipient vessels and donor nerves are available for neurotization, we recommend functional free gracilis transfer for hand reanimation.

This study has limitations, including a small cohort size, which makes reliable inferential statistics impossible. Selection bias is another limitation, as it is a series of cases, and the surgeon selected the surgical technique for hand reanimation based on clinical examination. However, no patient who underwent hand reanimation was excluded, reducing the bias. Considering that reanimation is an unusual surgery with precise indications, a retrospective study is an important tool for studying hand reanimation in total paralysis of the hand.

### Clinical significance

Biceps transfer to the finger flexors is a viable alternative for hand reanimation, offering reduced technical difficulties. Free-functioning muscle transfer for hand reanimation is a demanding surgery with expected good results when adequate donor nerves are available. Reconstructive microsurgeons can use both techniques as surgical options for hand reanimation.

## CONCLUSION

Biceps transfer to the finger flexors with tendon grafts is a viable alternative for hand reanimation in patients, offering fewer technical difficulties and reduced surgical team demands compared to free-functioning muscle transfer. However, functional free muscle flap is recommended as the first option whenever technically feasible, due to its potential for achieving greater final muscular strength in the finger flexors.
